# Effectiveness of a flipped classroom combined with team-based learning in standardized training of general practice residents: a randomized controlled trial

**DOI:** 10.3389/fmed.2026.1931590

**Published:** 2026-10-21

**Authors:** Meixia Xiao, Yashu Wang, Yujian Gao, Shengming Shi, Can Qian

**Affiliations:** Department of General Practice, First Affiliated Hospital of Huzhou Normal University, The First People’s Hospital of Huzhou, Huzhou, China

**Keywords:** clinical reasoning, flipped classroom, general practice residency training, randomized controlled trial, team-based learning

## Abstract

**Background:**

This study evaluated the effectiveness of a flipped classroom combined with team-based learning (TBL) training model in improving clinical reasoning, teamwork skills, self-directed learning ability, and teaching satisfaction among general practice residents in standardized training.

**Objective:**

This study aimed to: (1) compare theoretical knowledge mastery between the Flipped-TBL group and the traditional LBL group; (2) evaluate the effects of the Flipped-TBL model on residents’ clinical reasoning, teamwork skills, and self-directed learning ability; (3) examine differences in learning engagement, teaching satisfaction, and perceived stress between the two teaching models; and (4) provide an effective and innovative teaching approach for standardized general practice residency training.

**Methods:**

A 12-week randomized controlled trial was conducted with 56 general practice residents randomly assigned to either the FC-TBL group (*n* = 28) or traditional lecture-based learning (LBL) group (*n* = 28). The primary outcome was clinical reasoning ability, measured by the Lasater Clinical Judgment Rubric at baseline (T0), immediately post-intervention (T1), and four-week follow-up (T2). Secondary outcomes included theoretical knowledge, Mini-CEX, DOPS, and teaching satisfaction. Statistical significance was set at *p* < 0.05.

**Results:**

All 56 participants completed the study. The FC-TBL group demonstrated significantly greater improvement in clinical reasoning at T1 (14.68 ± 1.89 vs. 11.21 ± 1.76, *p* < 0.001) and T2 (13.96 ± 1.67 vs. 10.82 ± 1.58, *p* < 0.001) compared to the LBL group. Improvement from baseline to T1 was also significantly greater in the FC-TBL group (6.79 ± 1.45 vs. 3.17 ± 1.32, *p* < 0.001). The FC-TBL group significantly outperformed the LBL group across all secondary outcomes, including theoretical knowledge, all Mini-CEX and DOPS domains, teamwork skills, self-directed learning ability, learning engagement,and all teaching satisfaction dimensions (all *p* < 0.001). Perceived stress was transiently higher in the FC-TBL group at T1 (*p* < 0.001) but resolved by T2 (*p* = 0.892).

**Conclusions:**

The FC-TBL model is significantly more effective than traditional LBL in improving clinical reasoning, theoretical knowledge, clinical competency, procedural skills, self-directed learning ability, learning engagement, and teaching satisfaction among general practice residents. The transient increase in perceived stress resolved over time. This innovative model represents a promising pedagogical approach for standardized general practice residency training.

## 1 Introduction

The standardized training of general practitioners is a crucial component of post-graduation medical education and a strategic initiative in China's healthcare workforce planning (1). General practitioners serve as the “gatekeepers” of primary care, requiring not only solid biomedical knowledge but also strong clinical reasoning, teamwork, and lifelong learning skills (2). However, the current standardized training system in China has been criticized for its over-reliance on traditional lecture-based learning (LBL), in which residents passively receive information during didactic sessions with limited opportunities for active application, peer collaboration, or self-directed inquiry (3). Although LBL is efficient for transmitting factual knowledge, it falls short in fostering higher-order cognitive competencies that are essential for managing the complex, undifferentiated, and multifaceted biopsychosocial problems commonly encountered in primary care (4).

The flipped classroom (FC) has emerged as a promising alternative to traditional LBL, shifting foundational content delivery to the pre-class period and freeing in-class time for active, application-oriented learning (5). However, the mere inversion of class time does not guarantee effective learning; without a structured in-class pedagogical framework, sessions can devolve into unstructured group work or revert to mini-lectures (6). Team-based learning (TBL) provides such a structure, offering a highly organized, evidence-based strategy that promotes individual accountability, team collaboration (7). TBL has demonstrated positive effects on knowledge retention, clinical reasoning, and teamwork skills in medical education (8). Yet, TBL's effectiveness depends heavily on adequate pre-class preparation—a major challenge in busy residency programs where residents often struggle to complete lengthy pre-class readings.

The flipped classroom and TBL are not competing strategies but rather complementary pedagogies that address each other's limitations (9). FC ensures that learners acquire foundational knowledge through engaging, easily accessible pre-class video materials, thereby improving the quality of pre-class preparation that TBL requires. In turn, TBL provides a structured, accountable, and collaborative in-class framework that capitalizes on the flipped time, transforming it from passive lecture time into active application time (10).

Despite the theoretical promise of integrating FC and TBL, and preliminary evidence from undergraduate medical education settings (11, 12), no randomized controlled trial has systematically evaluated the effectiveness of the flipped classroom combined with TBL （FC-TBL**)** in the context of standardized general practice residency training. This gap is concerning, as general practice residents face unique learning demands—integrating knowledge across multiple disciplines, managing chronic conditions, and making decisions under uncertainty—that require precisely the competencies that FC-TBL targets. Therefore, this study aimed to evaluate the effectiveness of the FC-TBL model compared to traditional LBL in standardized training of general practice residents.

## 2 Materials and methods

### 2.1 Participants

A total of 56 participants were general practice residents undergoing standardized training at the Department of General Practice, First People's Hospital of Huzhou, from September 2023 to February 2026. All participants were medical graduates from various medical colleges and universities across China, all holding a bachelor's degree in clinical medicine and having passed the national medical licensing examination prior to enrollment in the residency program.

The inclusion criteria were as follows: (1) voluntary participation in this study and signing the informed consent form; (2) full-time undergraduate graduation with a bachelor's degree in clinical medicine; (3) no obvious physical or psychological abnormalities; and (4) no prior exposure to the flipped classroom or team-based learning (TBL) teaching models. The exclusion criteria were (1) involuntary participation in this study; (2) absence from more than 20% of the scheduled teaching sessions; and (3) failure to complete either the baseline or post-intervention assessments. All methods were performed in accordance with the Declaration of Helsinki. Finally, 100%participated in the trial. The study was approved by the Institutional Ethics Committee of the First People's Hospital of Huzhou City (approval No.2024HZYY-LL-SS-002). Written informed consent was obtained from all participants. The training curricula All responses were anonymized throughout the study. and rotation schedules implemented in this study adhere strictly to the Standardized Training Content and Standards for Resident Physicians (Trial)—General Practice Training Guidelines.

### 2.2 Randomization and allocation concealment

Participants were randomly assigned to either the FC-TBL group (experimental group) or the traditional lecture-based learning (LBL) group (control group) in a 1:1 ratio using a computer-generated randomization sequence prepared by an independent statistician not involved in recruitment, teaching, or outcome assessment. Allocation was concealed in sequentially numbered, opaque, sealed envelopes, which were opened by the study coordinator only after participants had completed all baseline assessments and signed the informed consent form. Due to the nature of the educational interventions, participants and instructors could not be blinded to group assignment; however, outcome assessors were fully blinded to group allocation for all performance-based assessments, with two trained assessors independently scoring each participant's clinical performance on-site.

### 2.3 Research design

This was a 12 week, two arm, parallel group superiority randomized controlled trial comparing the FC-TBL model against traditional LBL in a standardized general practice residency setting. All participants completed the same clinical rotation curriculum and case mix, with group assignment determining only the instructional strategy received during weekly two hour sessions. The 12-week intervention covered six clinical cases, with each case taught over approximately two weeks (two 2-hour sessions). The total instructional time was 24 contact hours per group. The FC-TBL strategy followed a four phase sequence: pre-class video-based preparation, in-class individual and team readiness assurance tests with immediate feedback, and team application exercises on complex cases. The LBL strategy consisted of instructor centered didactic lectures covering identical content without team activities. Outcomes were collected at baseline (T0), immediately post-intervention (T1), and at four week follow-up (T2). The primary outcome was clinical reasoning (Laseter Clinical Judgment Rubric). Secondary outcomes included theoretical knowledge (immediate and delayed), teamwork skills, self-directed learning ability, learning engagement, perceived stress, and teaching satisfaction. All performance assessments were scored on-site by two blinded, trained assessors.

#### 2.3.1 FC-TBL group (experimental group)-hypertension case

To illustrate the differences between the two teaching methods, the hypertension management case is presented below as a representative example. This case was taught during weeks 3–4 of the 12-week rotation. The same structure was followed for all six clinical cases (community-acquired pneumonia, hypertension, type 2 diabetes mellitus, heart failure, COPD, and acute kidney injury).

（1）**flipped preparation** (individual, 3–4 days before class). Residents were required to watch a pre-recorded video lecture (12 min) covering the foundational knowledge of hypertension, including: (1) definition and classification; (2) pathophysiology; (3)diagnostic criteria and risk stratification; (4) non-pharmacological and pharmacological treatment principles; (5) long term follow up in primary care. After watching the video, each resident completed an online pre-class quiz of 5 multiple choice questions. Completion was tracked by the instructor via a WeChat based learning platform.

（2）**Individual Readiness Assurance Test** (iRAT) ((in-class，individual, 10 min). The two hour in-class session began with a 10 question multiple choice test assessing the foundational knowledge covered in the pre-class video. Each resident completed the test individually using an electronic response system. Answers were recorded but not immediately disclosed.

（3）**Team Readiness Assurance Test** (tRAT) (in-class, team, 20 min). Immediately after the iRAT, the same 10 questions were administered again, but this time residents worked in small teams of 5–6 members. Teams discussed each question, reached consensus, and submitted their answers using a scratch card system that provided immediate feedback (correct/incorrect).

（4）**Team application exercise** (in-class, team, 40 min). After the readiness assurance process, teams engaged in a complex, authentic clinical case-Mr. Zhang, 65 years old, presents with a 5-year history of hypertension. His current medications include hydrochlorothiazide 25 mg daily. Today his BP is 162/98 mmHg. He reports poor medication adherence due to frequent urination at night. He has a 10-year history of type 2 diabetes (HbA1c 7.8%) and recently developed leg edema. Laboratory results show serum creatinine 1.3 mg/dL (eGFR 55 mL/min), potassium 3.2 mmol/L.

Teams were asked to: (1) identify the key problems (poor BP control, medication non-adherence, hypokalemia, possible diabetic nephropathy); (2) generate a differential diagnosis for leg edema; (3) propose a revised treatment plan; (4) develop a patient education strategy addressing medication adherence and lifestyle modifications; (5) propose a follow-up schedule. Each team presented their conclusions (5 min per team), followed by a 10 min instructor led debriefing comparing team responses with evidence based guidelines. After the session, residents completed a brief structured reflection log (5 min).

The detailed FC-TBL teaching process is shown in Figure 1.

**Figure 1:**
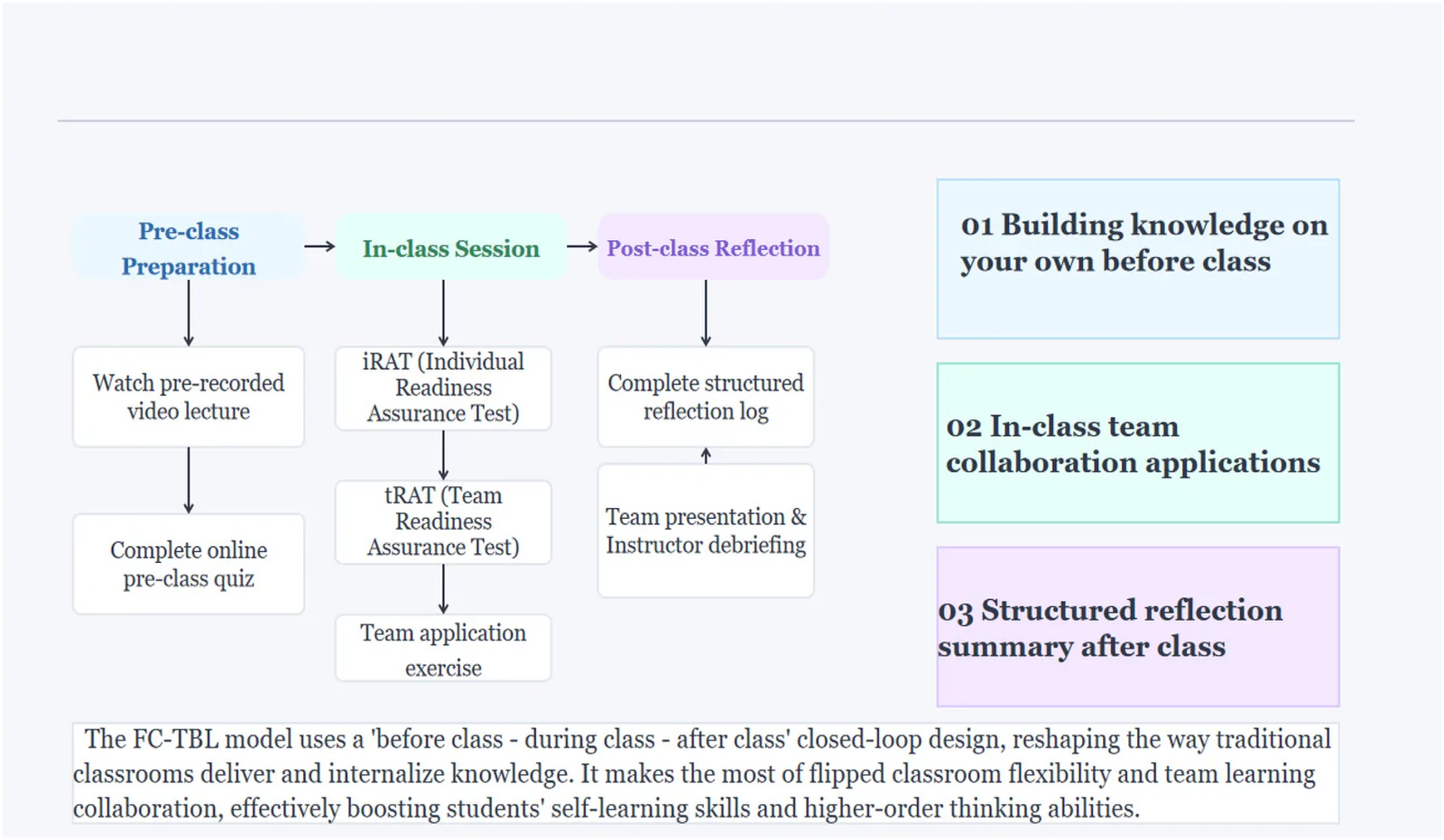
FC-TBL teaching model process. The FC-TBL model uses a 'before class-during class-after class' closed-loop design, reshaping the way traditional classrooms deliver and internalize knowledge. It makes the most of flipped classroom flexibility and team learning collaboration, effectively boosting students' self-learning skills and higher-order thinking abilities.

#### 2.3.2 LBL group (control group)-hypertension case

The LBL group received conventional lecture-based teaching for the same hypertension case. Before the session, residents were asked to read relevant textbook chapters and the guidelines (no video, no mandatory quiz).

During the two hour offline session, the instructor delivered a didactic lecture (90 min) covering: (1) definition, epidemiology, and risk factors; (2) pathophysiology;(3) diagnostic criteria and workup; (4) non pharmacological interventions; (5) pharmacological treatment; (6) management of special populations; and (7) long term follow up. The lecture included approximately 60 PowerPoint slides. After the lecture (30 min), the instructor demonstrated clinical skills: proper blood pressure measurement technique and interpretation of home BP monitoring logs. No student practice, no team activities, no iRAT/tRAT, no application exercise, and no structured feedback or reflection log were used. Students were encouraged to ask questions at the end of the session, but interaction was limited due to time constraints. The total contact time (2 h) and case content were identical to those of the FC-TBL group to ensure comparability.

### 2.4 Outcome indicators

The assessment of residents comprised five components: the Lasater Clinical Judgment Rubric (LCJR) for clinical reasoning assessment, a pre-class readiness test, a post-class summative test, the Mini Clinical Evaluation Exercise (Mini-CEX), and Direct Observation of Procedural Skills (DOPS).

The primary outcome was clinical reasoning ability, assessed using the Lasater Clinical Judgment Rubric (LCJR) at three time points: baseline (T0), immediately post-intervention (T1), and four-week follow-up (T2). The LCJR evaluates four dimensions of clinical judgment: noticing, interpreting, responding, and reflecting. Each dimension was scored on a 4-point scale (1 = beginning, 2 = developing, 3 = accomplished, 4 = exemplary), with total scores ranging from 4 to 16. Higher scores indicate superior clinical reasoning performance. Two trained and blinded assessors independently scored each resident's performance during the team application exercise and the objective structured clinical examination (OSCE) station, and the average score was used for analysis.

The Mini-CEX evaluated seven competency domains: medical interviewing, physical examination skills, clinical reasoning, organization and efficiency, doctor-patient communication, humanistic care, and overall clinical performance. Each domain was rated on a 9-point scale, where scores of 1–3 indicated “needs improvement,” 4–6 indicated “meets expectations,” and 7–9 indicated “exceeds expectations” (13). DOPS was employed to assess procedural competence, covering the following items: identification of indications and contraindications, informed consent process, pre-operative preparation, technical execution, communication with patients and staff, professionalism and respect for patient dignity, and overall procedural proficiency, with scoring criteria consistent with those of the Mini-CEX (14).

In addition to the knowledge tests, a structured questionnaire was administered to evaluate residents’ learning experiences and self-reported outcomes. Teaching satisfaction was assessed using a scale adapted from the Frontiers in Medicine study by Zhao et al. (15), which evaluated teaching satisfaction across four dimensions: teaching mode design, stimulation of learning interest, improvement of the learning process, and overall teaching satisfaction. Each dimension was rated on a 100-point scale, with higher scores indicating greater satisfaction.

Teamwork skills, self-directed learning ability, learning engagement, and perceived stress were assessed using a structured questionnaire adapted from validated instruments. Each item was rated on a 100-point scale, with higher scores indicating greater teamwork skills, self-directed learning ability, and learning engagement, and higher perceived stress, respectively.

### 2.5 Data evaluation and statistical analysis

Statistical analyses were performed using SPSS (version 26.0, IBM Corp., Armonk, NY, USA). Data normality was assessed using the Shapiro–Wilk test. Normally distributed continuous variables were expressed as mean ± SD, and compared using the independent samples t-test (between groups) or paired t-test (within groups). Non-normally distributed data were presented as median (IQR) and analyzed using the Mann–Whitney U test (between groups) or Wilcoxon signed-rank test (within groups). Categorical variables were summarized as frequencies and percentages, and compared using the chi-square test or Fisher's exact test as appropriate. The magnitude of improvement (post-intervention score minus pre-intervention score) for each outcome measure was calculated and compared between groups using the independent samples t-test or Mann–Whitney U test based on data distribution. Statistical significance was set at *p* < 0.05 (two-tailed). An *a priori* power analysis was performed to determine the required sample size. Assuming a moderate effect size (Cohen's d = 0.82) for the between-group difference in clinical reasoning scores, based on previous studies of flipped classroom interventions in medical education, a sample of 28 participants per group was required to achieve 80% power at a two-sided *α* of 0.05. The *post-hoc* power analysis, based on the observed between-group difference in improvement scores (Cohen's d = 2.59), confirmed that the achieved power exceeded 0.99.

## 3 Results

### 3.1 Participant characteristics

A total of 56 general practice residents were enrolled and randomly assigned to either the FC-TBL group (*n* = 28) or the LBL group (*n* = 28). All participants completed the 12-week intervention and all follow-up assessments, with no dropouts or exclusions. Baseline characteristics were well-balanced between the two groups, with no significant differences in age, gender, years of undergraduate education, National Medical Licensing Examination scores, or baseline clinical reasoning scores (all *p* > 0.05, Table 1).

**Table 1:** Baseline characteristics of participants in the FC-TBL and LBL groups.

Characteristic	FC-TBL group（*n* = 28)	LBL group（*n* = 28)	*χ^2^/t value*	*P value*
Sex，（Male/female）	12/16	13/15	0.071	0.790
Average age（years）	24.82 ± 1.42	25.07 ± 1.53	0.630	0.532
Undergraduate years	5.21 ± 0.42	5.18 ± 0.39	0.280	0.780
NMLE score	418.54 ± 32.67	421.39 ± 35.42	0.310	0.750
Baseline LCJR score	7.89 ± 1.45	8.04 ± 1.38	0.400	0.690

NMLE, national medical licensing examination; LCJR, Lasater clinical judgment rubric.

Sex was compared using the *χ^2^* test; continuous variables were compared using independent samples *t*-tests.

### 3.2 Clinical reasoning ability

The primary outcome was clinical reasoning ability, measured by the LCJR at T0, T1, and T2 (Table 2). No significant baseline difference was observed between groups (*p* = 0.692). At T1, the FC-TBL group scored significantly higher than the LBL group (*p* < 0.001), and this superiority was maintained at T2 (*p* < 0.001). The improvement from baseline to T1 was also significantly greater in the FC-TBL group (*p* < 0.001).

**Table 2:** Comparison of clinical reasoning scores (LCJR) between FC-TBL and LBL groups.

Time point	FC-TBL group（*n* = 28)	LBL group（*n* = 28)	*t value*	*P value*
T0 (Baseline)	7.89 ± 1.45	8.04 ± 1.38	0.40	0.69
T1(Immediate post)	14.68 ± 1.89	11.21 ± 1.76	7.10	< 0.001
T2 (four-week follow-up)	13.96 ± 1.67	10.82 ± 1.58	7.23	< 0.001
Improvement (T1–T0)	6.79 ± 1.45	3.17 ± 1.32	9.76	< 0.001

### 3.3 Mini-CEX scores

Clinical competency was evaluated using the Mini-CEX at T1 (immediately post-intervention). As shown in Table 3, the FC-TBL group demonstrated significantly higher scores across all seven Mini-CEX domains compared to the LBL group (all *p* < 0.001). These findings indicate that the FC-TBL model significantly enhanced residents’ overall clinical competency.

**Table 3:** Comparison of mini-CEX scores between FC-TBL and LBL groups.

Domain	FC-TBL group（*n* = 28)	LBL group（*n* = 28)	*t value*	*P value*
Medical interviewing	7.82 ± 0.86	6.14 ± 0.93	7.03	< 0.001
Physical examina tion skills	7.68 ± 0.91	5.96 ± 0.87	7.33	< 0.001
Clinical reasoning	8.04 ± 0.79	6.07 ± 0.94	8.51	< 0.001
Organization and efficiency	7.89 ± 0.82	6.21 ± 0.89	7.45	< 0.001
Doctor-patient communication	7.96 ± 0.78	6.32 ± 0.91	7.26	< 0.001
Humanistic care	7.93 ± 0.81	6.39 ± 0.94	6.66	< 0.001
Overall clinical performance	7.86 ± 0.84	6.07 ± 0.92	7.71	< 0.001

### 3.4 DOPS scores

DOPS was employed to assess procedural competence at T1. As presented in Table 4, the FC-TBL group demonstrated significantly higher scores across all seven DOPS domains compared to the LBL group (all *p* < 0.001), indicating that the FC-TBL model effectively enhanced residents’ procedural skills.

**Table 4:** Comparison of DOPS scores between FC-TBL and LBL groups.

Domain	FC-TBL group（*n* = 28)	LBL group（*n* = 28)	*t value*	*P value*
Indications and contraindications	8.11 ± 0.74	6.18 ± 0.89	8.83	< 0.001
Informed consent process	7.96 ± 0.82	6.21 ± 0.86	7.88	< 0.001
Pre-operative preparation	7.82 ± 0.86	5.96 ± 0.92	7.91	< 0.001
Technical execution	7.71 ± 0.89	5.89 ± 0.94	7.56	< 0.001
Communication with patients and staff	7.89 ± 0.83	6.14 ± 0.91	7.60	< 0.001
Professionalism and respect	8.04 ± 0.78	6.32 ± 0.89	7.75	< 0.001
Overall procedural proficiency	7.86 ± 0.81	6.04 ± 0.93	7.88	< 0.001

### 3.5 Teaching Satisfaction

Teaching satisfaction was assessed using a scale adapted from Zhao et al. (2024), which evaluated four dimensions on a 100-point scale. As shown in Table 5, the FC-TBL group reported significantly higher teaching satisfaction scores across all four dimensions compared to the LBL group (all *p* < 0.001), indicating that the FC-TBL model was well-received by residents.

**Table 5:** Comparison of teaching satisfaction scores between FC-TBL and LBL groups.

Dimension	FC-TBL Group (*n* = 28)	LBL Group (*n* = 28)	t-value	p-value
Teaching mode design	88.57 ± 6.34	72.14 ± 8.76	8.01	< 0.001
Stimulation of learning interest	87.86 ± 7.12	70.54 ± 9.23	7.86	< 0.001
Improvement of the learning process	86.79 ± 6.89	71.07 ± 8.54	7.59	< 0.001
Overall teaching satisfaction	89.29 ± 5.98	73.57 ± 8.92	7.81	< 0.001

### 3.6 Teamwork skills, self-directed learning ability, learning engagement, and perceived stress

As shown in Table 6, the FC-TBL group scored significantly higher than the LBL group in teamwork skills, self-directed learning ability, and learning engagement at both T1 and T2 (all *p* < 0.001). Perceived stress was transiently higher in the FC-TBL group at T1 (*p* < 0.001) but resolved by T2 (*p* = 0.892).

**Table 6:** Comparison of secondary outcomes between FC-TBL and LBL groups.

Outcome	Time	FC-TBL (*n* = 28)	LBL (*n* = 28)	t value	P value
Teamwork skills	T1	86.42 ± 6.78	72.15 ± 8.34	7.12	<0.001
	T2	84.97 ± 7.12	71.06 ± 8.91	6.84	<0.001
Self-directed learning	T1	85.73 ± 7.24	70.88 ± 9.16	6.93	<0.001
	T2	84.21 ± 7.56	70.14 ± 9.02	6.57	<0.001
Learning engagement	T1	87.15 ± 6.89	71.43 ± 8.77	7.38	<0.001
	T2	85.68 ± 7.03	70.92 ± 8.54	6.92	<0.001
Perceived stress	T1	62.34 ± 9.21	54.17 ± 8.76	3.48	<0.001
	T2	51.28 ± 8.43	50.96 ± 8.12	0.14	0.892

### 3.7 iRAT and tRAT scores in the FC-TBL group

The iRAT and tRAT were administered during each FC-TBL session. The mean iRAT score was 7.82 ± 1.24, and the mean tRAT score was 9.21 ± 0.87. The tRAT score was significantly higher than the iRAT score (mean difference: 1.39 ± 1.02, *t* = 7.21, *P* < 0.001), indicating that team discussion and consensus-building improved residents’ understanding of the pre-class material.

## 4 Discussion

This randomized controlled trial evaluated the effectiveness of a flipped classroom combined with team-based learning (FC-TBL) model in standardized training of general practice residents. Our findings demonstrate that the FC-TBL model significantly enhanced clinical reasoning ability, theoretical knowledge acquisition and retention, teamwork skills, self-directed learning ability, learning engagement, and teaching satisfaction compared to traditional lecture-based learning. Notably, the FC-TBL group also reported transiently higher perceived stress at immediate post-intervention, which resolved by four-week follow-up. These results suggest that the FC-TBL model represents an effective and well-received pedagogical approach for general practice residency training.

The most clinically meaningful finding of this study was the substantial improvement in clinical reasoning ability, as measured by the Lasater Clinical Judgment Rubric. The FC-TBL group demonstrated a mean improvement of 6.79 points from baseline to immediate post-intervention, compared to only 3.17 points in the LBL group (between-group difference in improvement: Cohen's d = 2.59), and this superiority was maintained at four-week follow-up. The between-group difference in total LCJR scores at T1 was also substantial (Cohen's d = 1.90). Clinical reasoning—the ability to gather and synthesize information, generate differential diagnoses, and make evidence-based decisions—is a cornerstone competency for general practitioners （^16^）.The FC-TBL model appears particularly well-suited to developing this competency（^17^）. The flipped preparation phase ensured that residents acquired foundational knowledge before class, while the in-class readiness assurance tests reinforced individual accountability. Critically, the team application exercises exposed residents to authentic, complex cases that required them to integrate knowledge across multiple domains, collaborate with peers, and articulate clinical reasoning under conditions that mirror real-world uncertainty. This structured sequence aligns with constructivist learning theory, which posits that knowledge is actively constructed through meaningful interaction with authentic problems and collaborative discourse.

The FC-TBL model also demonstrated significant advantages in fostering teamwork skills (T1: 86.42 ± 6.78 vs. 72.15 ± 8.34, *p* < 0.001) and self-directed learning ability(T1: 85.73 ± 7.24 vs. 70.88 ± 9.16, *p* < 0.001). Teamwork skills are essential in primary care, where general practitioners coordinate care across multidisciplinary teams, including nurses, pharmacists, social workers, and specialists （^18^）. The TBL component of our intervention—with its emphasis on team readiness assurance, consensus-building, and collaborative problem-solving—appears to have directly cultivated these competencies. The significantly higher self-directed learning ability scores in the FC-TBL group suggest that the pre-class video preparation and structured reflection logs fostered habits of independent inquiry and lifelong learning. This finding is particularly important given the rapidly evolving nature of medical knowledge and the need for general practitioners to continuously update their clinical practice. The transiently higher perceived stress in the FC-TBL group at T1 (62.34 ± 9.21 vs. 54.17 ± 8.76, *p* < 0.001) resolved by T2 (51.28 ± 8.43 vs. 50.96 ± 8.12, *p* = 0.892), suggesting that the additional workload was well-tolerated.

The iRAT and tRAT results provide insight into the mechanism underlying the FC-TBL model's effectiveness. The mean iRAT score of 7.82 indicated that residents generally completed the pre-class preparation and acquired foundational knowledge, but individual understanding was incomplete. The significantly higher tRAT score (9.21) demonstrated that team discussion and peer teaching effectively corrected misconceptions and deepened understanding. This collaborative learning process—where residents explain concepts to each other, defend their reasoning, and reach consensus—is a key mechanism through which TBL enhances knowledge acquisition and retention. These findings are consistent with previous research showing that the iRAT-to-tRAT sequence promotes both individual accountability and team learning.

Teaching satisfaction was assessed using a scale adapted from Zhao et al. （^15^）, which evaluated four dimensions on a 100-point scale. The FC-TBL group reported significantly higher satisfaction scores across all dimensions: teaching mode design (88.57 vs. 72.14), stimulation of learning interest (87.86 vs. 70.54), improvement of the learning process (86.79 vs. 71.07), and overall teaching satisfaction (89.29 vs. 73.57). These findings corroborate those of Feng et al. (10), who reported that online flipped classroom with TBL promoted learning activity in a clinical laboratory immunology class. Similarly, a mixed-methods study on FC-TBL in molecular biology laboratory teaching found that the FC-TBL group achieved significantly higher satisfaction rates, with 76.4% of students reporting that the approach stimulated their motivation to learn independently, compared to only 27.8% in the LBL group (19). Residents in our study particularly valued the interactive, application-oriented nature of the FC-TBL sessions, which contrasted sharply with the passive experience of traditional lectures. However, the FC-TBL group also reported a higher perceived workload increase, reflecting the more demanding nature of pre-class preparation and team activities. Nevertheless, the overwhelming majority expressed acceptance of the teaching model and satisfaction with learning outcomes, suggesting that the additional workload was well-tolerated and perceived as worthwhile.

The superior performance of the FC-TBL group on both Mini-CEX and DOPS assessments further reinforces the model's effectiveness. The Mini-CEX, which evaluates clinical competency across seven domains, and DOPS, which assesses procedural skills, are validated tools for formative and summative assessment in residency training. The FC-TBL group outperformed the LBL group across all domains, with the largest differences observed in clinical reasoning, overall clinical performance, and physical examination skills. These findings align with Ding et al (11)., who reported that flipped classroom combined with case- and team-based learning in residency training enhanced students’ clinical competence. They also resonate with Shuai et al. (12), who demonstrated the application of flipped classroom combined with TBL in orthopedic clinical teaching improved teaching effectiveness.

Several factors may explain why the FC-TBL model was particularly effective in this general practice residency context. First, general practice residents face unique learning demands that require integration of knowledge across multiple disciplines, management of chronic conditions, and decision-making under uncertainty (20). The FC-TBL model's emphasis on complex, authentic case application directly addresses these demands. Second, the model addresses two critical challenges in residency education: the difficulty of ensuring adequate pre-class preparation (addressed by engaging video materials and mandatory quizzes) and the need for structured, accountable in-class activities (addressed by the TBL framework). Third, the model capitalizes on the social and collaborative dimensions of learning, which are particularly valuable in primary care settings where team-based practice is the norm (21).

### 4.1 Limitations

Several limitations of this study should be acknowledged. First, this was a single-center study with a relatively small sample size of 56 participants, which may limit the generalizability of our findings to other training settings with different resident populations or institutional resources. Although the *post-hoc* power analysis indicated adequate statistical power for the primary outcome, larger multicenter studies are needed to confirm these findings. Second, due to the nature of the educational interventions, participants and instructors could not be blinded to group assignment. While outcome assessors were blinded for all performance-based assessments, the lack of participant blinding may have introduced performance bias. Third, the relatively short follow-up period of four weeks limited our ability to assess long-term retention of clinical skills and their translation into independent practice. Fourth, although the Lasater Clinical Judgment Rubric is a validated tool for assessing clinical reasoning, it relies on observer ratings, which are inherently subjective despite the use of two trained and blinded assessors. Future studies should incorporate objective structured clinical examinations (OSCEs) or standardized patient encounters to further validate these findings. Finally, the teaching satisfaction questionnaire, while adapted from a validated instrument, was modified for this specific context and may benefit from further psychometric validation.

### 4.2 Implications for practice

Despite these limitations, our findings have several practical implications for general practice residency training. First, the FC-TBL model offers a feasible and effective alternative to traditional lecture-based teaching that can be implemented within existing curricular structures. The 12-week intervention duration and two-hour weekly sessions are comparable to typical residency training schedules, suggesting that the model can be integrated without major curricular redesign. Second, the model addresses the pressing need to develop clinical reasoning and teamwork competencies that are essential for primary care practice but are inadequately addressed by traditional LBL. Third, the positive teaching satisfaction scores indicate that residents are receptive to this active learning approach, which may enhance motivation and engagement. Fourth, the transient increase in perceived stress should be anticipated and addressed through orientation sessions that prepare residents for the active learning demands and emphasize the benefits of the approach.

### 4.3 Future research directions

Future research should explore the long-term impact of FC-TBL training on clinical performance and patient outcomes in independent practice. Large-scale, multi-center randomized controlled trials with extended follow-up periods are warranted to confirm and expand upon our findings. Additionally, qualitative studies exploring residents’ experiences and perceptions of the FC-TBL model could provide valuable insights into the mechanisms underlying its effectiveness. Comparative studies examining the relative contributions of the flipped classroom and TBL components would help elucidate which elements are most critical for improving different outcomes. Finally, cost-effectiveness analyses would inform decisions about resource allocation for implementing this model across training programs.

## 5 Conclusion

In conclusion, this randomized controlled trial demonstrates that the flipped classroom combined with team-based learning model is significantly more effective than traditional lecture-based learning in improving clinical reasoning, theoretical knowledge, teamwork skills, self-directed learning ability, learning engagement, and teaching satisfaction among general practice residents. While the model is associated with transiently higher perceived stress, this resolves over time and is outweighed by the substantial educational benefits. The FC-TBL model represents a promising pedagogical approach for standardized general practice residency training and may contribute to the development of competent, collaborative, and lifelong-learning-oriented primary care physicians.

## Data Availability

The raw data supporting the conclusions of this article will be made available by the authors, without undue reservation.
